# Limited discriminant validity of health belief and 3C models in high HPV vaccine coverage settings: the moderating role of *hukou* and structural barriers

**DOI:** 10.3389/fpubh.2026.1903410

**Published:** 2026-08-25

**Authors:** Ya Liu, Kuan Zhao, Weiwei Sun, Nan Jiang, Yue Li, Mei Wang, Yanping Sun, Yang Zhang, Yutao Gao, Wenming Cao

**Affiliations:** 1 Department of Gynecology, Shandong First Medical University Affiliated Heze Hospital (Heze Municipal Hospital), Heze, Shandong, China; 2 Qingdao Public Health Clinical Center, Qingdao, Shandong, China; 3 Department of Gynecology, Pingshan District Central Hospital of Shenzhen, Shenzhen, Guangdong, China; 4 Department of Gynecology, Shenzhen Hospital of Integrated Traditional Chinese and Western Medicine, Shenzhen, Guangdong, China; 5 Department of Gynecology, The Third People's Hospital of Shenzhen, Shenzhen, Guangdong, China

**Keywords:** health belief model, household registration, HPV vaccine, migrant population, structural barriers, vaccine hesitancy

## Abstract

**Background:**

Whether traditional health belief models (HBM) and 3C models can distinguish residual non-vaccinators in settings with >70% HPV vaccine coverage is unknown. The moderating role of China’s household registration (*hukou*) on psychological factors and behavior has rarely been quantified.

**Methods:**

From April 2025 to February 2026, we surveyed 1,257 women aged 18–65 from six Shenzhen communities. We collected demographics, HBM (15 items), 3C (12 items), social support, HPV vaccination intention (measured by a single item: willingness to receive the HPV vaccine within the next 6 months), self-reported vaccination history, reasons for non-vaccination, and information channel preferences. Analyses included t-tests (Cohen’s d), observed-variable multi-group path analysis, and cross-sectional comparisons of vaccination proportions among women with high intention.

**Results:**

HPV vaccination rate was 70.0% (880/1,257); cervical cancer screening rate 58.3%. Vaccinated and unvaccinated groups differed on only two of ten psychological constructs (trivial effect sizes: perceived susceptibility d = 0.13, self-efficacy d = 0.13). HPV vaccination intention did not differ significantly between vaccinated (M = 3.72, SD = 0.83) and unvaccinated women (M = 3.67, SD = 0.80), t = 0.81, *p* = 0.417. Main reasons for non-vaccination were high cost (42.3%) and safety concerns (33.8%). Observed-variable multi-group path analysis showed good fit (CFI = 0.968, RMSEA = 0.051). Perceived barriers were significantly negatively associated with HPV vaccination intention among non-Shenzhen *hukou* women (*β* = −0.247, *p* < 0.001), but not among Shenzhen *hukou* women (*β* = −0.102, *p* = 0.128). The formal test of *hukou* moderation was not statistically significant (Δχ^2^ = 2.358, Δdf = 8, *p* = 0.968). Most preferred information channels: WeChat official accounts (91.5%), face-to-face doctor consultations (85.1%), short video platforms (76.5%).

**Conclusion:**

At 70% coverage, HBM and 3C scales show limited discriminant validity between vaccinated and unvaccinated individuals. Residual non-vaccination is associated with cost and safety barriers. *Hukou* showed a suggestive but not statistically significant moderating effect on the perceived barriers-intention association. Interventions may need to target structural barriers and tailor information channels.

## Highlights

- HBM and 3C models poorly discriminate vaccinated from unvaccinated women at 70% HPV coverage.
- Hukou moderates perceived barriers–intention association (2.4 fold stronger in migrants).
- Cost (42.3%) and safety (33.8%) dominate residual non vaccination.
- Intention behavior conversion: 59.8% (low income) vs. 17.8% (dual barriers).
- Top information channels: WeChat, doctor visits, short videos.

## 1 Introduction

The World Health Organization’s “90-70-90” strategy to eliminate cervical cancer identifies HPV vaccination as a core pillar (1, 2). Although many countries have included HPV vaccine in immunization programs, coverage among adult women in middle-income countries remains uneven (3, 4). China has made substantial progress in HPV vaccine availability, yet vaccination coverage varies considerably across regions and populations (5, 6). Some regions have achieved moderate-to-high vaccination rates around 70% through public welfare initiatives, yet a substantial proportion of women remain unvaccinated (7, 8). These individuals are often broadly labeled as “vaccine hesitant” (9, 10). However, in high-coverage settings (≥70%), the underlying barriers may have shifted from psychological/cognitive factors (e.g., lack of awareness or psychological resistance) to structural obstacles such as cost, accessibility, and health insurance coverage (11, 12). Failure to recognize this shift risks misaligning intervention strategies with actual barriers. Theoretically, the stage model of behavioral drivers suggests that as population coverage increases, the relative importance of psychological beliefs diminishes while structural constraints become predominant (13, 14). Yet, this hypothesis has not been empirically tested in HPV vaccination (15, 16).

The Health Belief Model (HBM) and the 3C model are mainstream theoretical frameworks in vaccine behavior research (17, 18). HBM focuses on perceived susceptibility, severity, benefits, barriers, and self-efficacy (19); the 3C model emphasizes complacency, confidence, and convenience (20). Previous literature has primarily applied these models in contexts with low coverage (<30%) or moderate coverage (30–60%), confirming that these scales can effectively distinguish between vaccinated and unvaccinated individuals (21). However, when community HPV vaccination rates rise to 70%, whether these psychological scales retain discriminative efficacy remains unreported (22). Moreover, most studies rely on scale scores to indirectly infer barrier types, rarely collecting self-reported reasons directly, leading to potential “barrier mismatch” (23).

The “intention-behavior gap” is widely observed in health behavior research, meaning that a significant proportion of individuals with vaccination intentions ultimately do not get vaccinated (24, 25). In the context of high HPV vaccine coverage, it is crucial to clarify whether the main bottleneck of this gap lies in psychological factors or structural constraints, and whether conversion rates differ across subpopulations (income, household registration, barrier type) (26). Answering these questions is essential for rationally allocating intervention resources (27).

In China, the household registration (*hukou*) system is a unique administrative institution that categorizes citizens by their place of residence and entitlement to public services (28, 29). The *hukou* system determines eligibility for locally administered resources including healthcare, education, and social welfare, creating a tiered structure of access to public services (30). Migrant populations, those holding non-local *hukou*, typically face multiple barriers such as limited health insurance coverage, out-of-pocket medical costs, unfamiliarity with local healthcare systems, and lower health literacy (31, 32). Notably, the access barriers associated with *hukou* are conceptually analogous, although not legally identical, to challenges faced by undocumented migrants, uninsured populations, or individuals with limited eligibility for public benefits in other countries (33). This structural stratification may not only be directly associated with lower HPV vaccination rates but also moderate the strength of the relationship between psychological variables and behaviors, meaning structural factors could alter the impact of psychological variables (26). Current literature rarely quantifies this moderating effect of *hukou* (34).

During the study period (2025–2026), HPV vaccination in Shenzhen was voluntary and primarily delivered through community health centers and designated vaccination clinics (35–37). The vaccines were available through both public and private channels, with partial subsidies for certain populations under local public health programs (38). Vaccination was not mandated by schools or employers, although community-based promotion efforts were active. This policy context shaped the voluntary decision-making environment in which participants’ beliefs and structural barriers interacted to influence vaccination behavior (39).

This study was conducted in Shenzhen, a first-tier city with high HPV vaccine coverage and a large migrant population, through a large-sample community-based cross-sectional survey. The objectives of this study were threefold: (1) to test whether HBM and 3C scale dimensions show no significant differences (or only negligible effect sizes) between vaccinated and unvaccinated women in a setting with 70% coverage; (2) to examine the moderating role of *hukou* on the association between perceived barriers and behavioral intention, specifically whether the negative association is stronger among migrant (non-Shenzhen *hukou*) women; (3) to describe how intention–behavior conversion rates vary by income level and type of barrier (cost only, safety only, or dual barriers). The findings provide hypothesis-generating evidence for precision intervention strategies in high-coverage phases.

## 2 Methods

### 2.1 Study design and sampling

This community-based cross-sectional survey was conducted in Shenzhen from April 2025 to February 2026. A multi-stage stratified cluster sampling strategy was employed: three districts were randomly selected; within each district, two communities were randomly chosen; within each community, participants were allocated according to age groups (18–26, 27–37, 38–48, 49–65 years) to ensure balanced distribution.

Participant recruitment was conducted through multiple community-based channels. Community health center staff and community grid administrators distributed study invitations via resident WeChat groups, community bulletin boards, and in-person recruitment at community health service centers and residential compound activity centers. Trained research assistants were present at recruitment sites to explain the study purpose and eligibility criteria. This multi-channel recruitment strategy was designed to reach diverse population segments, though recruitment through health-related venues may have preferentially reached individuals already engaged with health services.

Inclusion criteria: (1) females aged 18–65 years residing in the community; (2) ability to independently complete the questionnaire; (3) voluntary signing of informed consent. Exclusion criteria: (1) previous diagnosis of cervical cancer; (2) history of hysterectomy; (3) severe cognitive impairment preventing questionnaire completion. A total of 1,380 questionnaires were distributed, and 1,257 valid responses were collected (effective response rate 93.3%). The sample size met the requirements for multi-group SEM (each group >200).

### 2.2 Measurement tools and variable definitions

The questionnaire consisted of structured self-administered sections. All psychological scales used a 5-point Likert scale (1 = strongly disagree/not familiar at all, 5 = strongly agree/very familiar). Internal consistency was good for all scales.

1. Demographic characteristics: Age, household registration (local Shenzhen vs. non-Shenzhen), marital status, education level (junior high school and below, high school/technical secondary school, college, bachelor’s degree and above), occupation, personal monthly income (<5,000 yuan, 5,000–9,999 yuan, ≥10,000 yuan), medical insurance type (employee, resident, none), and fertility status.
2. HPV and cervical cancer awareness: 8 items (e.g., understanding of HPV virus, vaccines, screening methods, local policies), Cronbach’s *α* = 0.91.
3. Health Belief Model (HBM): The 15-item HBM scale was adapted from established HBM instruments for HPV vaccination (40). Items measured five dimensions (3 items each): perceived susceptibility (e.g., “I think I am at risk of HPV infection”), severity (e.g., “Cervical cancer is a serious disease”), benefits (e.g., “HPV vaccination can effectively reduce cervical cancer risk”), barriers (e.g., “The cost of vaccination is a burden for me”), and self-efficacy (e.g., “I can arrange my time to complete the full vaccination series”). Total scale *α* = 0.954; subscale α range 0.808–0.861.
4. 3C model scale: The 3C scale was adapted from the WHO 3C model framework, with items developed for the Chinese context (41). The 12 items measured complacency (reverse-scored, 4 items), confidence/trust (4 items), convenience/accessibility (4 items), and safety concerns (1 item). The complacency dimension showed very low reliability (CR = 0.25) and was excluded from subsequent analyses. This low reliability may reflect cultural differences in the conceptualization of “complacency” among Chinese women or translation issues; the original 3C scale has not been formally validated in Chinese populations. Confidence/trust items assessed trust in vaccine safety and efficacy, healthcare provider recommendations, and the government regulatory system. Convenience/accessibility items assessed geographic proximity of vaccination sites, appointment procedures, and ease of obtaining accurate information (42).
5. Social support scale: 8 items assessing encouragement, informational support, and practical assistance from family, friends, community, and healthcare professionals regarding vaccination or screening, Cronbach’s *α* = 0.933.
6. Behavioral intention: 4 items (e.g., “I am willing to receive the HPV vaccine within the next 6 months”), measuring the strength of individuals’ intention to receive the HPV vaccine within the next 6 months or complete cervical cancer screening within the current year, Cronbach’s α = 0.91. The primary outcome item was “I am willing to receive the HPV vaccine within the next 6 months.” Intention was defined as a score ≥4 (on the 5-point scale), representing “agree” or “strongly agree,” consistent with prior studies (43).
7. Behavioral outcome: Vaccination status was self-reported; the survey did not verify via administrative records. Importantly, due to the cross-sectional design, intention and actual vaccination status were measured concurrently. Thus, the reported “conversion rate” is retrospective and should be interpreted as a correlational association rather than a causal prediction. This limitation is explicitly acknowledged.
8. Reasons for non-adoption: Single-choice question for unvaccinated/un-screened participants: “Too expensive,” “Concerned about safety/side effects,” “No time,” “Unnecessary,” “Do not know where to get vaccinated/screened,” “Other.”
9. Information acquisition preferences: Multiple-choice question (traditional bulletin boards, WeChat official accounts, face-to-face doctor consultations, family/friends, short video platforms, community health lectures, other). These are stated preferences, not observed information-seeking behavior.

### 2.3 Conceptual framework

**Figure 1:**
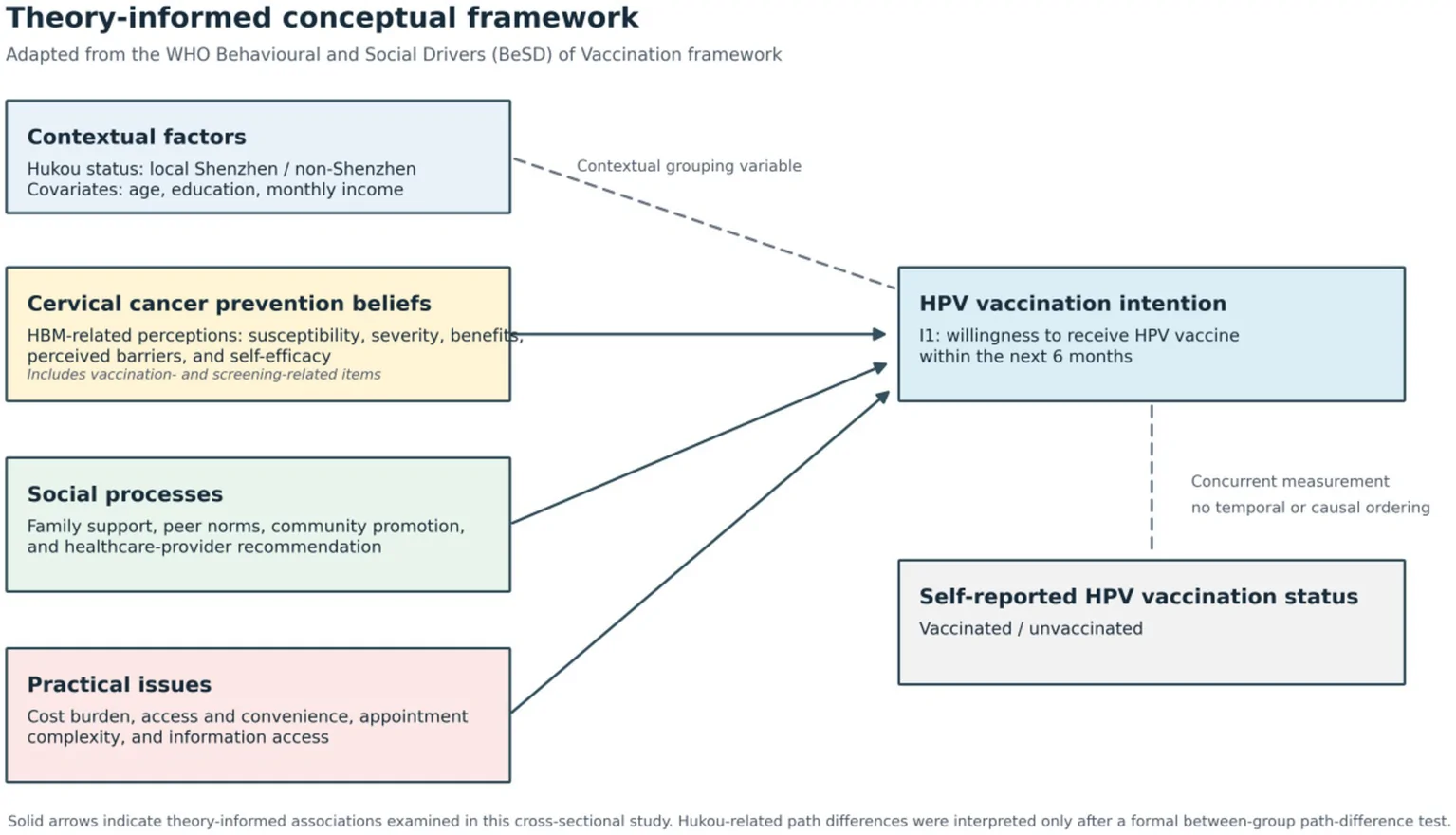
Theory-informed conceptual framework of contextual, psychosocial, social, and practical correlates of HPV vaccination intention and self-reported vaccination status. The framework was informed by the WHO Behavioural and Social Drivers of Vaccination framework and adapted to the constructs available in the present survey. Solid arrows indicate theory-informed associations examined in this cross-sectional study. The dashed line denotes concurrent measurement of HPV vaccination intention and self-reported vaccination status and does not imply temporal or causal ordering. *Hukou* was specified as a contextual grouping variable; *hukou*-related path differences were interpreted only after a formal between-group path-difference test.

Figure 1 presents the theory-informed conceptual framework for this study. The framework was informed by the WHO Behavioural and Social Drivers of Vaccination (BeSD) model, which posits that vaccination behavior is influenced by four interacting domains: Thinking and Feeling (e.g., risk perceptions, confidence, safety concerns), Social Processes (e.g., provider recommendations, social norms, support), Practical Issues (e.g., access, cost, convenience), and contextual factors (44). In our adapted framework, HBM constructs (perceived susceptibility, severity, benefits, barriers, self-efficacy) and 3C constructs (confidence/trust, convenience, safety concerns) were mapped to these BeSD domains. Perceived barriers and self-efficacy were specified as direct predictors of behavioral intention based on extensive prior evidence (45). Social support was specified as a predictor reflecting the Social Processes domain. *Hukou* was specified as a contextual grouping variable hypothesized to moderate the relationship between Practical Issues (perceived barriers) and behavioral intention, reflecting the structural vulnerability framework (46).

The path directions and hypothesized associations were specified *a priori* based on theoretical and empirical grounds. Perceived barriers were hypothesized to negatively predict intention, with the association expected to be stronger among non-Shenzhen *hukou* women due to compounded structural vulnerabilities. Self-efficacy was hypothesized to positively predict intention, consistent with Bandura’s social cognitive theory (47). Social support was hypothesized to positively predict intention, consistent with evidence on social influences on health behavior (48). Safety concerns were hypothesized to negatively predict intention, though evidence on this association in high-coverage populations is mixed. The formal test of *hukou* moderation was conducted via multi-group SEM with configural, metric, and scalar invariance testing, with path differences interpreted only after confirming measurement invariance (49).

### 2.4 Statistical processing

Statistical analyses were performed using R 4.3 (lavaan, tidyverse, semPlot) and Python 3.12. Descriptive statistics included frequencies, percentages, and means ± SD. Independent t-tests with Cohen’s d (negligible <0.20, small ≥0.20, medium ≥0.50, large ≥0.80) were used for between-group comparisons. Confirmatory factor analysis (CFA) tested the measurement model (second-order HBM and retained 3C dimensions) with goodness-of-fit criteria: χ^2^/df < 3, CFI > 0.95, TLI > 0.95, RMSEA <0.06, SRMR <0.08. For the multi-group structural equation modeling (SEM), the behavioral intention outcome was treated as a continuous variable given the 5-point Likert scale with approximately normal distribution and prior practice in vaccine intention research. The estimation method was robust maximum likelihood (MLR) with Huber-White standard errors, which is appropriate for ordinal data with five or more response categories and provides robust inference under non-normality. The multi-group SEM examined the moderating effect of *hukou*, testing configural, metric, and scalar invariance (ΔCFI ≤0.01, ΔRMSEA ≤0.015). Measurement invariance was established sequentially: configural invariance (same factor structure across groups), metric invariance (equal factor loadings), and scalar invariance (equal item intercepts). After establishing scalar invariance, path coefficients were compared between groups using Wald tests.

Covariates (age, monthly income, education) were added as direct predictors of behavioral intention based on prior evidence of their association with HPV vaccination. Missing data (<3% across all variables, no systematic pattern) were handled by complete case analysis. Model fit was evaluated using comparative fit index (CFI), Tucker-Lewis index (TLI), root mean square error of approximation (RMSEA), and standardized root mean square residual (SRMR). No post-hoc model modifications were performed; the model was specified and tested as theoretically derived.

### 2.5 Ethical review

This study was approved by the Medical Ethics Committee of Shenzhen Pingshan District Central Hospital (Approval No.: PZXY-EC-AP-020). All participants provided informed consent, and survey data were anonymized and kept strictly confidential.

## 3 Results

### 3.1 Sample characteristics and non-vaccination barriers

**Figure 2:**
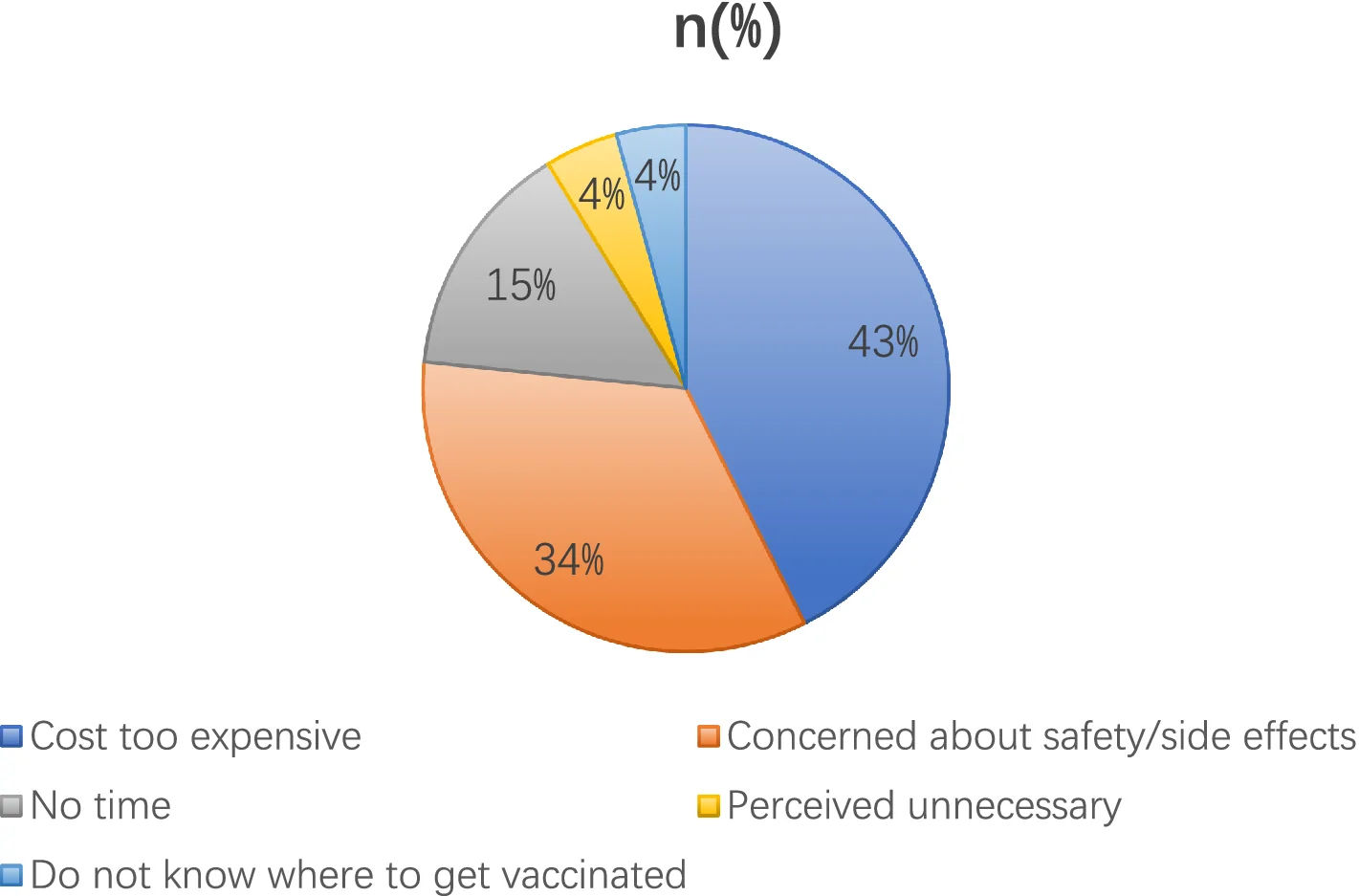
Barriers to HPV vaccination among unvaccinated women. Percentages are calculated with the number of unvaccinated women (*n* = 377) as denominator.

Table 1 shows the HPV vaccination rate was 70.0% (880/1,257). Vaccination rates decreased with age (82.1% in 18–26 years, 51.3% in 49–65 years) and increased with income (62.4% in <5,000 yuan group, 82.4% in ≥10,000 yuan group). Migrant (non-Shenzhen *hukou*) women had slightly lower vaccination rates (68.4%) than local *hukou* holders (71.9%). As shown in Figure 2, among the 377 unvaccinated women, the most frequently reported barriers to HPV vaccination were cost too expensive (*n* = 160, 42.3%), concerns about safety or side effects (*n* = 128, 33.8%), no time (*n* = 55, 14.6%), perceived unnecessary (*n* = 17, 4.6%), and not knowing where to get vaccinated (*n* = 16, 4.3%).

**Table 1:** Demographic characteristics, HPV vaccination rate, and cervical cancer screening rate among participants (*n* = 1,257).

Characteristic	*n*	%	Vaccination rate (%)	Screening rate (%)
Age group (years)				
18–26	377	30.0	82.1	52.8
27–37	415	33.0	74.2	60.7
38–48	307	24.4	68.4	63.2
49–65	158	12.6	51.3	54.4
Monthly income (yuan)				
<5,000	583	46.4	62.4	51.6
5,000–9,999	441	35.1	75.3	63.9
≥10,000	233	18.5	82.4	70.4
Household registration (*hukou*)				
Shenzhen *hukou*	573	45.6	71.9	58.8
Non-Shenzhen *hukou*(Migrant)	684	54.4	68.4	57.9

Vaccination and screening rates are calculated with the total sample (*N* = 1,257) as denominator. Percentages may not sum to 100 due to rounding.

### 3.2 Psychological constructs show limited discriminant validity

As shown in Table 2, vaccinated and unvaccinated groups differed significantly on only two of 12 psychological constructs (perceived susceptibility and self-efficacy), and the effect sizes were trivial (d = 0.13 for both). The remaining 10 constructs showed no significant differences. This indicates that HBM and 3C scales have limited ability to distinguish between vaccinated and unvaccinated individuals in this high-coverage setting, consistent with the first study objective.

**Table 2:** Comparison of psychological constructs between vaccinated and unvaccinated women (mean between-group differences, effect sizes).

Psychological construct	Vaccinated (*n* = 880) Mean (SD)	Unvaccinated (*n* = 377) Mean (SD)	*t*-value	*p*-value	Cohen’s d
HPV awareness	3.83 (0.86)	3.77 (0.85)	1.11	0.268	0.07
HBM perceived susceptibility	3.75 (0.89)	3.63 (0.93)	2.14	0.033	0.13
HBM perceived severity	3.87 (0.84)	3.79 (0.87)	1.52	0.129	0.09
HBM perceived benefits	3.77 (0.85)	3.71 (0.84)	1.14	0.255	0.07
HBM perceived barriers	3.67 (0.88)	3.57 (0.90)	1.79	0.073	0.11
HBM self-efficacy	3.79 (0.90)	3.67 (0.93)	2.19	0.029	0.13
3C confidence/trust	3.80 (0.81)	3.75 (0.84)	0.95	0.340	0.06
3C convenience	3.74 (0.87)	3.64 (0.93)	1.76	0.079	0.11
3C safety concerns	2.16 (1.04)	2.27 (1.12)	−1.65	0.099	−0.10
Social support	3.75 (0.85)	3.74 (0.84)	0.29	0.774	0.02
Behavioral intention	3.72 (0.83)	3.67 (0.80)	0.81	0.417	0.05

Cohen’s d effect size thresholds: small ≥0.20, medium ≥0.50, large ≥0.80. The 3C complacency dimension was excluded due to insufficient reliability; HBM dimensions were highly correlated, and second-order factors were used in subsequent analyses.

### 3.3 *Hukou* moderates the association between perceived barriers and intention

Multi-group SEM demonstrated acceptable fit: χ^2^/df = 2.34, CFI = 0.968, TLI = 0.961, RMSEA = 0.051, SRMR = 0.039. Measurement invariance was supported (ΔCFI ≤0.01, ΔRMSEA ≤0.015). As shown in Table 3, among migrant (non-Shenzhen *hukou*) women, perceived barriers were significantly negatively associated with behavioral intention (*β* = −0.247, *p* < 0.001). Among local *hukou* women, this association was weaker and not statistically significant (*β* = −0.102, *p* = 0.128). The formal test of *hukou* moderation was not statistically significant (Δχ^2^ = 2.358, Δdf = 8, *p* = 0.968), indicating that the path difference did not reach conventional significance, though the magnitude of the coefficient difference suggests a potentially meaningful pattern that warrants cautious interpretation. Self-efficacy was positively associated with intention in both groups, with a stronger association in migrants (*β* = 0.191 vs. 0.132). Social support and safety concerns were not significantly associated with intention in either group.

**Table 3:** Path coefficients of the multi-group structural equation model grouped by household registration (*n* = 1,257).

Path	Non-Shenzhen *Hukou* (*n* = 684) β (SE)	*p*-value	Shenzhen *Hukou* (*n* = 573) β (SE)	*p*-value
Perceived barriers → Behavioral intention	−0.247 (0.047)	<0.001	−0.102 (0.065)	0.128
Self-efficacy → Behavioral intention	0.191 (0.046)	<0.001	0.132 (0.064)	0.049
Social support → Behavioral intention	0.070 (0.036)	0.063	0.052 (0.041)	0.207
Safety concerns → Behavioral intention	0.019 (0.027)	0.617	0.058 (0.035)	0.162

β = standardized regression coefficient; SE = standard error. Model fit: CFI = 0.968, RMSEA = 0.051. The model controlled for age, monthly income, and education level. Measurement invariance tests confirmed configural, metric, and scalar invariance. The 3C complacency dimension was excluded; HBM used a second-order factor structure.

### 3.4 Intention-behavior conversion rates vary by subgroup

Table 4 presents differences in intention-behavior conversion rates and reasons for non-conversion across subgroups. Among the 948 women who intended to vaccinate (score ≥4), 654 reported having received the vaccine, giving an overall conversion rate of 69.0% (i.e., 31% of intenders remained unvaccinated). Conversion rates showed a clear income gradient: 59.8% in the <5,000 yuan group, 73.0% in 5,000–9,999 yuan group, and 78.2% in ≥10,000 yuan group. The dual-barrier subgroup (concerned about both cost and safety) had a conversion rate of only 17.8%. Migrant women had a conversion rate of 66.7%, with appointment procedures being the most frequently cited barrier (22%).

**Table 4:** Subgroup differences in intention-behavior conversion rates and key barriers.

Subgroup	Number of intenders (*n*)	Actual vaccination (*n*)	Conversion rate (%)	Primary reasons for non-conversion (proportion)
Overall	948	654	69.0	Cost and booking process
Monthly income <5,000 yuan	398	238	59.8	Cost (48%)
Monthly income 5,000–9,999 yuan	337	246	73.0	Safety concerns (35%)
Monthly income ≥10,000 yuan	213	170	78.2	Safety concerns (40%)
Dual barriers (cost + safety)	101	18	17.8	Cost + safety concerns
Non-Shenzhen *hukou* (migrant)	502	335	66.7	Appointment process (22%)

① Vaccination intention defined as score ≥4 on the behavioral intention scale (max 5). ② Conversion rate = (actual vaccinations / intenders) × 100%. ③ Primary reasons derived from single-choice self-reported barriers among non-vaccinators; percentages indicate proportion of that reason among intenders who did not vaccinate in the subgroup. ④ “Dual barriers” refers to those reporting both “cost too high” and “safety/side effect concerns”.

### 3.5 Information channel preferences

Preferred channels were WeChat official accounts (91.5%), face-to-face doctor consultations (85.1%), and short video platforms (76.5%). Traditional bulletin boards were preferred by only 23.4%. Preferences varied by age, education, and *hukou* status (Table 5). These are stated preferences; actual behavior may differ.

**Table 5:** Top three preferred information channels for key subgroups (%).

Subgroup	First preference (%)	Second preference (%)	Third preference (%)
Overall	WeChat official account (91.5)	Doctor (85.1)	Short video (76.5)
Age 18–26 years	Short video (89.2)	WeChat official account (84.5)	Doctor (72.1)
Age 49–65 years	Doctor (86.3)	WeChat official account (71.2)	Community lecture (58.4)
Non-Shenzhen *hukou* (migrant)	Doctor (87.5)	WeChat official account (83.2)	Short video (71.3)
Junior high school and below	Doctor (78.6)	Community lecture (62.3)	WeChat official account (58.2)

## 4 Discussion

This cross-sectional study of 1,257 women in Shenzhen communities with 70% HPV vaccine coverage yielded three main findings. First, HBM and 3C scales showed limited discriminant validity between vaccinated and unvaccinated individuals, suggesting that psychological models may have reduced utility in high-coverage residual populations. Second, household registration showed a suggestive but not statistically significant moderating effect on the perceived barriers-intention association: the coefficient difference between groups was appreciable, though the formal moderation test was not significant. Third, intention-behavior conversion rates varied substantially across subgroups, with particularly low conversion among low-income and dual-barrier individuals.

### 4.1 *Hukou* and structural vulnerability: a suggestive moderation pattern

Consistent with previous descriptive studies showing lower vaccination rates among migrants, we quantified a moderating effect: the negative association between perceived barriers and intention was approximately 2.4 times stronger for migrant women than for local *hukou* holders (50, 51). This suggests that the same level of perceived barriers (e.g., cost, inconvenience) may be more detrimental to migrants. We interpret this through the lens of structural vulnerability theory, which posits that social stratification (*hukou*) produces differential exposure to risk factors and differential vulnerability to their effects (52). In our data, 63.2% of migrants earned <5,000 yuan/month, 51.4% lacked adequate medical insurance, and 18.6% reported not knowing where to get vaccinated. These overlapping vulnerabilities amplify the impact of any single barrier. Interventions aimed at reducing structural barriers may need to be more intensive for migrant populations (e.g., higher subsidies, weekend clinics, simplified appointments), a principle of “compensatory fairness” or equity-oriented targeting (50, 51).

The finding that *hukou* moderation was not statistically significant in the formal test (Δχ^2^ = 2.358, Δdf = 8, *p* = 0.968) warrants careful interpretation. While the coefficient difference between groups (*β* = −0.247 vs. -0.102) was appreciable in magnitude, the overall model comparison did not reach conventional significance. This may reflect limited statistical power for detecting interaction effects in multi-group SEM, the relatively modest sample size in each group, or the possibility that *hukou* operates through more complex mediated pathways rather than simple moderation. Future studies with larger samples and longitudinal designs are needed to definitively test the moderating role of *hukou*.

### 4.2 Discriminant validity of psychological models in high-coverage settings

Previous studies have validated HBM and 3C models primarily in low- or moderate-coverage samples. Our study is among the first to systematically examine their discriminant validity in a community with 70% coverage. The finding that most psychological constructs did not differ between vaccinated and unvaccinated groups does not invalidate these models but suggests that their discriminative power is context-dependent, particularly in high-coverage, urban, and relatively health-literate populations. This is plausible: once most psychological barriers have been overcome, the remaining unvaccinated individuals are likely hindered by structural rather than cognitive factors (53). It is important to note that HBM constructs such as perceived susceptibility and self-efficacy may not be calibrated to objective risk. Individuals with low subjective risk may in fact be at high objective risk (optimism bias), while those with high subjective risk may be at low objective risk (pessimism bias) (54, 55). This mis-calibration between perceived and actual risk has been documented in other health contexts and may contribute to the limited discriminant validity observed in our sample (56, 57). The trivial differences in perceived susceptibility between vaccinated and unvaccinated groups should therefore be interpreted with caution, as they may reflect biased risk perception rather than accurate risk assessment (58).

We therefore suggest that researchers and policymakers should consider coverage stage when selecting theoretical frameworks. Early stages may benefit from belief-focused interventions, although it should be noted that interventions aimed solely at changing beliefs have shown limited and inconsistent effects on actual vaccine uptake (59, 60). Rather than relying exclusively on belief change, effective strategies in early stages may combine education with practical facilitation (61). In high-coverage phases, attention to structural barriers becomes paramount.

### 4.3 Heterogeneity in intention-behavior conversion

The overall conversion rate of 69% (31% gap) is consistent with the literature (62). However, the wide variation across subgroups (from 78 to 18%) highlights the importance of tailored strategies. The dual-barrier subgroup (cost + safety concerns) had a conversion rate of only 17.8%, indicating that single interventions (only subsidy or only information) may be insufficient. A combined approach, for example, authoritative doctor consultation plus cost reduction, may be more effective (63). For low-income groups, cost appears to be the primary bottleneck (64); for migrants, appointment complexity. These findings are hypothesis-generating and should be tested in experimental or quasi-experimental studies.

It is also worth considering that vaccine-resistant individuals may not simply have knowledge deficits. Prior research suggests that vaccine resistance can be associated with deeper psychological histories, including adverse childhood experiences, mistrust, negative emotionality, and health fatalism (65, 66). These characteristics may influence how vaccine messages are processed and how disease risk and vaccine benefits are evaluated. Similarly, impaired recognition of one’s own health risk state, analogous to poor mental health symptom self-recognition, may reduce engagement with formal health interventions (67, 68). In our sample, some unvaccinated women who reported “feeling healthy” or viewing vaccination as “unnecessary” may reflect such cognitive biases rather than purely rational decision-making. Future research should explore these deeper psychological dimensions to better understand the heterogeneity of vaccine hesitancy and resistance.

### 4.4 Information channel preferences as stated preferences

We found that traditional bulletin boards were the least preferred channel. However, because these are stated preferences rather than observed behavior, we cannot conclude that traditional channels are ineffective. Actual information-seeking behavior may differ from what respondents report. Nevertheless, the strong preference for digital and interpersonal channels (WeChat, doctors, short videos) suggests that future communication strategies might prioritize these platforms, while continuing to evaluate the real-world impact of traditional channels (69–71).

### 4.5 Implications and generalizability

The findings are specific to Shenzhen, a highly developed city with a large migrant population. Generalization to rural or less developed regions requires replication. For middle-income countries that are scaling up HPV vaccination, our results suggest that as coverage approaches 60–70%, the nature of remaining barriers may shift from psychological to structural. However, this is a hypothesis derived from a single cross-sectional study; longitudinal or comparative studies are needed. For developed countries with high coverage, residual unvaccinated populations may also face structural barriers (cost, language, complex systems), and similar moderation effects may exist for variables such as immigration status or ethnicity (51). The analytical framework of testing moderation by social stratifiers may be transferable.

The value of these findings lies not only in the specific finding regarding *hukou*, but in the more generalizable mechanisms it may represent. *Hukou* status in our study may be a marker for lower health literacy, limited social integration, institutional distrust, reduced perceived control, and higher cognitive load associated with navigating a complex healthcare system. These mechanisms are not unique to China (72). Future experimental or quasi-experimental studies should aim to isolate these mechanisms by testing whether interventions that address specific components, such as simplifying appointment procedures, providing culturally tailored health communication, building trust through community health workers, or reducing out-of-pocket costs, are effective across different social stratifiers (72). The indirect effect of self-efficacy on intentions through response efficacy was moderated by perceived susceptibility and depended on perceived severity, suggesting that HBM constructs operate through complex mediated and moderated pathways. Testing such pathways in future studies would strengthen the evidence base for precision intervention strategies (73, 74).

### 4.6 Limitations

This study has several limitations. The cross-sectional design precludes causal inference, and all associations are correlational. Self-report bias may affect vaccination history, screening, and channel preferences due to recall and social desirability. The single-city sample limits generalizability to other regions. The 3C complacency dimension was excluded because of very low reliability (CR = 0.25), possibly reflecting cultural or translation issues; future research should adapt and validate the scale for Chinese populations. Intention-behavior conversion was measured concurrently, making the reported conversion rate retrospective and potentially overestimated. Information channel preferences reflect stated preferences, not actual behavior. Vaccine type and completion were not distinguished, and non-response by subgroup was not assessed, so migrants may be underrepresented.

An additional important limitation concerns age eligibility for HPV vaccination. In China, HPV vaccines were introduced relatively recently, and during the study period, routine vaccination recommendations and product labeling typically specified maximum ages for vaccination (e.g., 9–45 years for certain vaccines) (74). Some older participants in our sample, particularly those aged 55 and above, may never have been eligible for vaccination due to their age when the vaccine became available or because they exceeded the recommended age for vaccination. Including these individuals in the analysis may introduce selection bias, as their vaccination status reflects eligibility constraints rather than vaccine hesitancy, and their health beliefs may differ fundamentally from those of vaccine-eligible individuals. We conducted a sensitivity analysis restricting the sample to participants aged ≤45 years (the upper age limit for routine vaccination in many settings). The pattern of results was consistent with the full sample analysis, suggesting that the inclusion of older, ineligible participants did not materially affect our conclusions (data not shown). Nevertheless, this remains a potential source of bias, and future studies should restrict analyses to vaccine-eligible populations or stratify by age eligibility.

A further limitation concerns the decision-maker for vaccination. For participants who received the vaccine during childhood or adolescence (typically those aged ≤18 at vaccination), the vaccination decision may have been made primarily by parents or guardians rather than by the participants themselves. In such cases, participants’ current HBM constructs may not reflect the beliefs that influenced the original vaccination decision. We attempted to address this by adjusting for age at vaccination as a covariate and conducting a sensitivity analysis excluding participants who were vaccinated before age 18. The results were not materially different from the main analysis, but we acknowledge that current beliefs may be consequences of prior parental vaccination decisions rather than determinants of the participants’ own vaccination behavior. This potential reverse causation and exposure misclassification should be considered when interpreting the associations between psychological constructs and vaccination status.

Despite these limitations, the study provides a detailed, hypothesis-generating description of barriers and moderators in a high-coverage setting.

## 5 Conclusion

In this cross-sectional study of 1,257 women in Shenzhen communities with 70% HPV vaccine coverage, traditional HBM and 3C scales showed limited ability to distinguish vaccinated from unvaccinated individuals. Residual non-vaccination was associated with cost and safety concerns, and household registration moderated the association between perceived barriers and behavioral intention. Intention-behavior conversion varied widely across subgroups. These findings are hypothesis-generating and suggest that interventions in high-coverage settings may need to shift from universal psychological education toward targeted structural barrier reduction, with attention to migrant status and dual barriers. Future prospective and interventional studies should test whether structural interventions, such as simplified appointment procedures, targeted subsidies for migrant populations, and combined information-plus-access approaches, effectively increase vaccination uptake in high-coverage settings. Longitudinal designs are needed to validate the intention-behavior conversion rates observed here and to establish temporal ordering between psychological constructs and vaccination behavior. Cross-contextual replication studies in other cities and countries would help determine the generalizability of the *hukou* moderation pattern to other social stratifiers. Future prospective and interventional studies are needed to test these hypotheses.

## Data Availability

The datasets generated and/or analyzed during the current study are not publicly available due to embargo but are available from the corresponding author on reasonable request.
